# Meyerson’s Phenomenon Surrounding a Seborrheic Keratosis: A Case Report

**DOI:** 10.7759/cureus.101080

**Published:** 2026-01-08

**Authors:** Emily T Huynh, Travis C Frantz, David S Kirwin, W. Hugh Lyford

**Affiliations:** 1 Division of Dermatology, Pacific Northwest University of Health Sciences College of Osteopathic Medicine, Yakima, USA; 2 Department of Dermatology, Naval Medical Center San Diego, San Diego, USA

**Keywords:** annular lesion, eczematous halo, halo dermatitis, meyerson's phenomenon, reactive skin disorder, seborrheic keratosis

## Abstract

Meyerson’s phenomenon (MP) is a rare immune-mediated local eczematous reaction that surrounds a pre-existing lesion, including (but not limited to) melanocytic nevi, vascular malformations, nevus sebaceus, and melanoma. We report a case of MP surrounding a seborrheic keratosis that had been non-reactive for the previous 25 years. The exact pathophysiology and antigenic triggers remain unknown. Topical steroids are commonly used to treat MP. However, removal of the inciting lesion may also provide resolution.

## Introduction

Meyerson’s phenomenon (MP), otherwise known as “halo dermatitis,” is a relatively uncommon immune-mediated presentation that was originally described in 1971 as a localized eczematous reaction surrounding a pre-existing melanocytic nevus [1]. This inflammatory reaction pattern has been described most commonly with melanocytic lesions and sporadically in a variety of other cutaneous lesions, including vascular malformations, nevus sebaceus, and malignant melanoma [2,3]. To our knowledge, only five cases of MP involving a seborrheic keratosis have been reported in the English literature [4-6].

Despite being a known entity, MP can present a diagnostic challenge to the clinician as it may appear morphologically similar to annular diseases, infections, or malignancy. Histopathologic correlation can aid in establishing the correct diagnosis.

MP lesions are histologically characterized by variable spongiosis, acanthosis, occasional parakeratosis, and a superficial infiltrate consisting of lymphocytes and eosinophils [7]. The pathophysiology of MP involves the local immune response, although the specific antigenic stimulus remains to be elucidated [8]. Treatment of MP typically includes topical steroids or removal of the inciting central lesion.

## Case presentation

**Figure 1:**
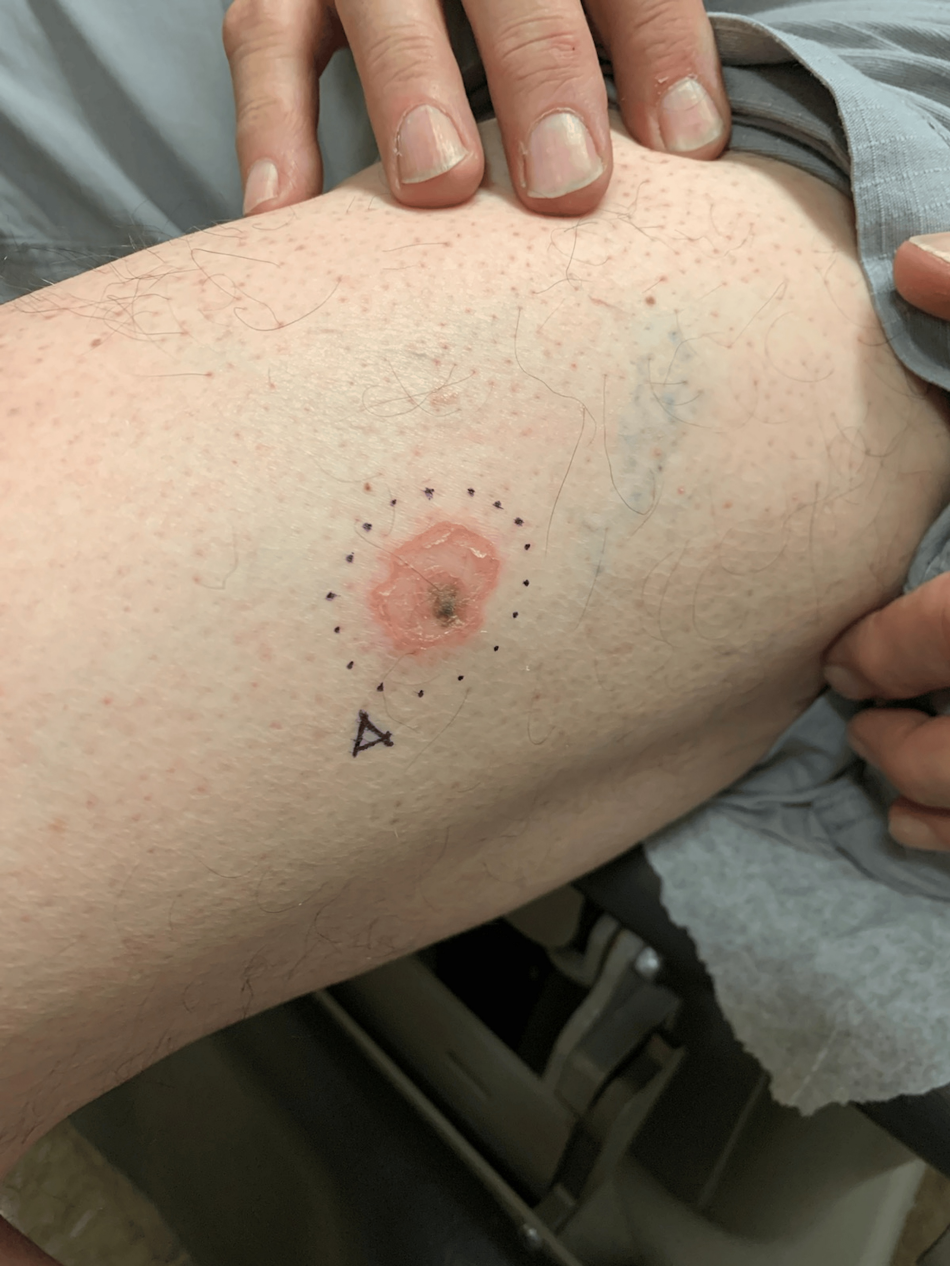
Erythematous annular plaque with a peripheral yellow scale, surrounding an eccentric tan papule on the left lateral thigh

A 71-year-old man presented to the dermatology clinic in San Diego County with a 2.2 cm mildly pruritic, well-circumscribed, erythematous annular plaque on the left lateral thigh of one month’s duration. The plaque demonstrated a peripheral yellow scale with mild central clearing (Figure 1). The patient reported that the erythematous ring had expanded gradually around an unremarkable tan papule, which had been present for over 25 years. The patient had no relevant past medical history, recent travel history, or other similar-appearing lesions. No treatments had been attempted prior to the initial evaluation.

**Figure 2:**
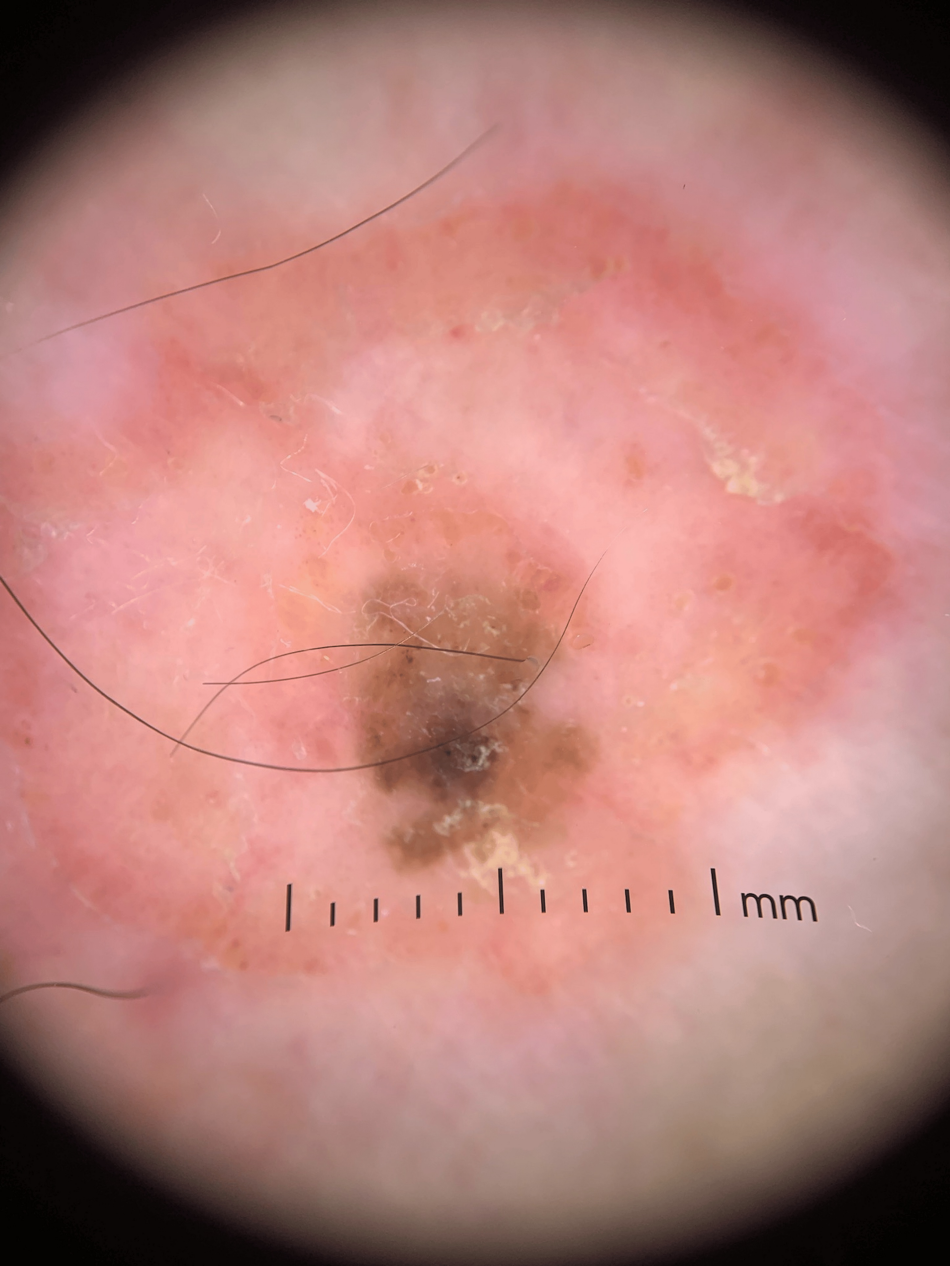
Dermoscopic view demonstrating an irregularly shaped tan papule with absent pigment network, comedo-like openings, and a central focal tan area suggestive of an irritated seborrheic keratosis

**Figure 3:**
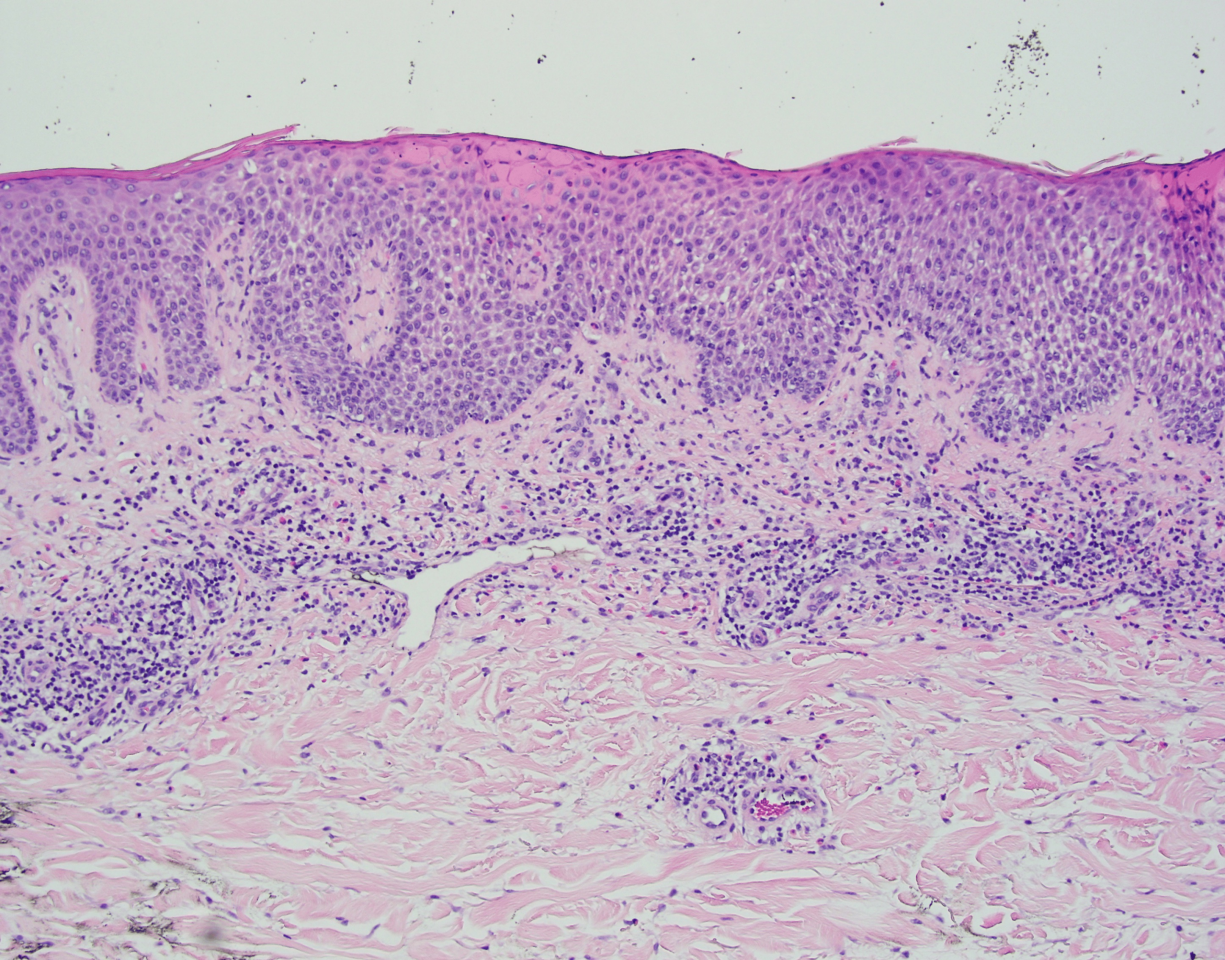
H&E, original magnification ×10: marked spongiosis of the epidermis and perivascular lymphohistiocytic infiltrates with scattered eosinophils H&E: hematoxylin and eosin

Dermoscopy of the tan papule revealed an irregularly shaped papule with comedo-like openings, an absent pigment network, and a central focal uniformly tan area suggestive of a seborrheic keratosis (Figure 2). A potassium hydroxide preparation was obtained and was negative for fungal elements. A shave biopsy was performed, removing the middle one-third of the lesion, including the entire tan papule. Histology revealed epidermal acanthosis with horn cyst formation at the lowest power. At higher power, prominent epidermal spongiosis and perivascular lymphohistiocytic infiltrate with scattered eosinophils were observed (Figure 3). Finally, there was an absence of nuclear pleomorphism, eccentric nucleoli, or atypical mitotic figures. The clinical examination, timing of plaque development around a pre-existing lesion, and histopathology supported a diagnosis of MP involving a seborrheic keratosis.

**Figure 4:**
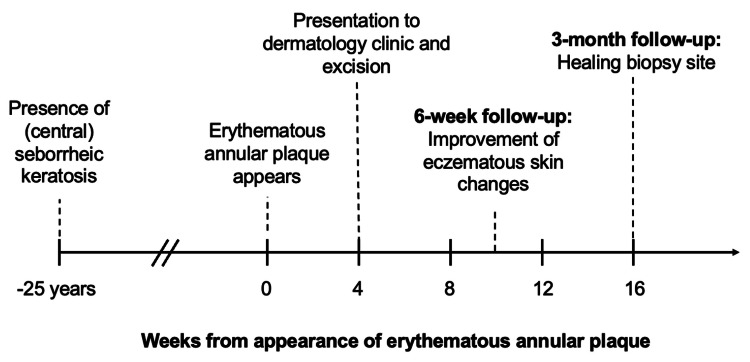
Timeline of clinical course

A follow-up examination six weeks following the removal of the seborrheic keratosis showed marked improvement of the eczematous skin changes. Follow-up at three months demonstrated a well-healing biopsy site (Figure 4). The eczematous reaction self-resolved without further intervention, suggesting an antigenic stimulus from the previously quiescent seborrheic keratosis.

## Discussion

This case highlights the rare condition of MP in a patient with no significant medical history. To our knowledge, this is the sixth reported case of MP around a seborrheic keratosis, and the condition may be underdiagnosed.

Similar to the current study, previously published cases of MP around seborrheic keratoses demonstrated a slight predominance in men and patients above the age of 50 (Table 1). This is likely due to the higher incidence of seborrheic keratoses in elderly individuals in general, although two cases of MP around seborrheic keratoses were reported in patients under 40 years of age. This contrasts with MP around melanocytic nevi, which tends to occur in individuals younger than 30 years of age [9]. There was no clear link in this case, nor in the literature, between the history of atopy and the development of MP around a seborrheic keratosis.

**Table 1:** Summary of five previously published cases of Meyerson’s phenomenon around a seborrheic keratosis *: irritated seborrheic keratosis, **: seborrheic keratosis and Meyerson’s phenomenon diagnosed at the same time

Literature	Age (years)	Sex	Site	History of atopy or hypersensitivity	Time between the appearance of the central lesion and halo dermatitis	Treatment
Tegner et al. (1989) [4]	78	Female	Trunk	Unknown	Unknown	Topical corticosteroid
Tegner et al. (1989*) [4]	36	Female	Lower extremity	Unknown	3 weeks	Excision
Rosen et al. (1990) [6]	Mid-20s	Male	Trunk	No	Many years	Excision
Rosen et al. (1990) [6]	57	Male	Trunk	Yes, asthma	Not applicable**	Excision
Dawn and Burden (2001*) [5]	65	Male	Lower extremity	No	Not applicable**	Topical corticosteroid

The differential diagnosis for MP includes a variety of conditions, which clinically present as annular plaques, including erythema annulare centrifugum (EAC), tinea corporis, cutaneous lichen planus, and granuloma annulare. On histology, both EAC and MP show perivascular lymphocytic infiltrates, spongiosis, and parakeratosis [10]. The most common location is the proximal extremities or the trunk [10]. However, EAC arises de novo, not typically in association with a nevus. Furthermore, EAC is distinguished by its “trailing scale” and papillary dermal edema, which were absent in this case [10]. EAC further lacks the eosinophils that are a key finding in MP [7,10]. This patient presented in the summer in the warm climate of California, but the lack of fungal elements on potassium hydroxide preparations made dermatophyte infection, such as tinea corporis, much less likely. Annular cutaneous lichen planus clinically has white reticular lines (Wickham striae) rather than scale and is characterized by vacuolar interface change [11]. A final differential considered was granuloma annulare, an inflammatory granulomatous condition most commonly found on the extremities. Unlike MP, pruritus and scaling are typically absent. Granuloma annulare’s distinct histology consists of a triad with the hallmark presence of mucin deposition within the dermis, palisading histiocytes, and lymphocytes surrounding a focus of collagen necrobiosis, which were not present here [12].

The antigenic trigger inducing MP remains unclear. Case reports have suggested risk factors including interferon immunotherapy, laser therapy (for a central nevus flammeus), and ultraviolet exposure [13,14]. In our case, MP appeared spontaneously and completely resolved after the removal of the central seborrheic keratosis, suggesting a molecular change in the lesion. Since the seborrheic keratosis had been previously unremarkable, it was reasonable to remove it to rule out malignant transformation.

While MP is an uncommon reactive process, histologic examination is crucial because rapid changes, including inflammation, scaling, irregular borders, and pruritus, can mimic malignant changes. Malignant transformation of seborrheic keratosis is exceedingly rare but well-documented. A retrospective analysis of 23,000 histopathologic examinations of clinically apparent seborrheic keratoses found that 11.9% were histologically diagnosed as basal cell carcinoma, 3.4% as squamous cell carcinomas, and 1.01% as malignant melanomas [15]. Dermoscopy and histopathology can differentiate the benign inflammatory changes of MP from true malignancy.

## Conclusions

The patient presented in this case experienced an inflammatory annular reaction surrounding a seborrheic keratosis that had been previously quiescent for decades. It remains unclear what triggered this presumed lack of self-tolerance. A skin biopsy may be indicated for erythematous annular lesions that appear around pre-existing skin lesions, as there is a broad differential. Additionally, central lesions within Meyerson’s phenomena can represent malignancy in some rare cases. Clinical and histopathologic recognition of this entity can aid prompt treatment with either removal (as in this case) or topical steroids and reassurance for the patient.
